# Platycodin D inhibits platelet function and thrombus formation through inducing internalization of platelet glycoprotein receptors

**DOI:** 10.1186/s12967-018-1688-z

**Published:** 2018-11-15

**Authors:** Qi Luo, Guangyu Wei, Xiaoqing Wu, Kai Tang, Mengdi Xu, Yulu Wu, Yun Liu, Xiaoqian Li, Zengtian Sun, Wen Ju, Kunming Qi, Chong Chen, Zhiling Yan, Hai Cheng, Feng Zhu, Zhenyu Li, Lingyu Zeng, Kailin Xu, Jianlin Qiao

**Affiliations:** 1 Blood Diseases Institute, Xuzhou Medical University, 84 West Huaihai Road, Xuzhou, 221002 China; 2 Department of Hematology, The Affiliated Hospital of Xuzhou Medical University, 99 West Huaihai Rd, Quanshan District, Xuzhou, 221002 China; 3 Key Laboratory of Bone Marrow Stem Cell, Xuzhou, Jiangsu China

**Keywords:** Platycodin D, Platelet, Glycoprotein receptors, Internalization, Hemostasis, Arterial thrombosis

## Abstract

**Background:**

Platycodin D (PD) is one of the major bioactive components of the roots of *Platycodon grandiflorum* and possesses multiple biological and pharmacological properties, such as antiviral, anti-inflammatory, and anti-cancer activities. However, whether it affects platelet function remains unclear. This study aims to evaluate the role of PD in platelet function and thrombus formation.

**Methods:**

Platelets were treated with PD followed by measuring platelet aggregation, activation, spreading, clot retraction, expression of glycoprotein receptors. Moreover, mice platelets were treated with PD and infused into wild-type mice for analysis of in vivo hemostasis and arterial thrombosis.

**Results:**

Platycodin D treatment significantly inhibited platelet aggregation in response to collagen, ADP, arachidonic acid and epinephrine, reduced platelet P-selectin expression, integrin α_IIb_β_3_ activation, spreading on fibrinogen as well as clot retraction, accompanied with decreased phosphorylation of Syk and PLCγ2 in collagen-related peptide or thrombin-stimulated platelets. Moreover, PD-treated mice platelets presented significantly impaired in vivo hemostasis and arterial thrombus formation. Interestingly, PD induced internalization of glycoprotein receptors α_IIb_β_3_, GPIbα and GPVI. However, GM6001, cytochalasin D, BAPTA-AM and wortmannin did not prevent PD-induced internalization of receptors.

**Conclusions:**

Our study demonstrates that PD inhibits platelet aggregation, activation and impairs hemostasis and arterial thrombosis, suggesting it might be a potent anti-thrombotic drug.

## Background

Platelets play an important role in thrombosis and hemostasis. At sites of vascular injury, platelets roll, adhere and firmly attach to the sub-endothelial matrix by membrane receptors, glycoprotein (GP)VI and GPIba through recognition of exposed collagen and von Willebrand factor (VWF) [1, 2]. Engagement of platelet receptors will trigger intracellular signaling transduction, leading to activation of platelet integrin α_IIb_β_3_ (inside-out signaling), from a low affinity to a high affinity, which binds to fibrinogen, fibronectin or VWF and mediates platelet aggregation and thrombus formation [3, 4]. Meanwhile, ligands binding to α_IIb_β_3_ also induce a series of intracellular signaling events (outside-in signaling), resulting in tyrosine phosphorylation of signaling proteins, including c-Src, spleen tyrosine kinase (Syk), phospholipase Cγ2 (PLCγ2), which initiates downstream platelet responses, such as granule secretion, platelet spreading, clot retraction as well as thrombus stabilization [5, 6].

Platycodin D, a triterpenoid saponin, is one of the major bioactive components of the roots of *Platycodon grandiflorum,* a traditional Chinese medicinal herb [7, 8]. It is also called Chinese balloon flower or common balloon flower in English [9]. As a Traditional Chinese medicine, *P. grandiflorum* has been widely used for the treatment of various diseases for thousands of years. The main active ingredients include triterpenoid saponin, flavanoid, phenolic compounds and fatty acids. Several monomers of *P. grandiflorum* have been identified, including platycodin A, B, C, D, D2, D3, and polygalacin D, D2 [9]. Previous studies have demonstrated that platycodin D possesses multiple biological and pharmacological properties, such as anti-nociceptive [10], antiviral [11], anti-inflammatory [12], anti-cancer [13], immunomodulatory [14, 15], and hepatoprotective activities [8]. Moreover, platycodin D could be a potential approach to treat obesity as it can inhibit lipogenesis in 3T3-L1 cells and modulate fat accumulation in obese mice [16]. Furthermore, it has also been reported to exert anti-atherosclerotic effect, possibly through increasing NO concentration, reducing the oxidized low-density lipoprotein-induced cell adhesion molecule expression in endothelial cells and the endothelial adhesion to monocytes [17]. Considering the broad spectrum activities on cell biological behaviors, whether platycodin D affects platelet function remains poorly understood.

In this study, through incubating platelets with different concentrations of platycodin D, we investigated the effect of platycodin D on platelet aggregation, activation, spreading as well as clot retraction. In addition, the effect of platycodin D on hemostasis and thrombosis in vivo was also evaluated.

## Materials and methods

### Reagents

Platycodin D (PD) was purchased from Fluka (Steinheim, Germany) with a purity ≥ 99%. Collagen, adenosine diphosphate (ADP), arachidonic acid and epinephrine were from Helena laboratories (Beaumont, Texas, USA). Thrombin was purchased from Sigma-Aldrich (St. Louis, MO, USA). FITC-conjugated mouse anti-human CD41a and PAC-1 antibody were from BD Biosciences (San Jose, CA, USA) and Becton–Dickinson (San Jose, CA, USA) respectively. PE-conjugated anti-human/mouse CD62p (P-Selectin) and anti-human Glycoprotein VI purified antibody were purchased from eBioscience (San Diego, CA, USA). FITC-conjugated anti-CD42b antibody was from Abcam (Cambridge, MA, USA). FITC-conjugated goat anti-mouse IgG was purchased from ZSGB-BIO (Beijing, China). β-actin antibody and anti-rabbit IgG (HRP-linked) antibody were purchased from Cell Signaling Technology (Danvers, MA, USA).

### Animals

The care and use of animals were in accordance with the guidelines of Xuzhou Medical University. All experimental procedures involving animals were complied with ARRIVE guidelines and approved by the Ethic Committee of Xuzhou Medical University. C57BL/6 mice, aged 8–10 weeks and weighted 24–28 g were purchased from SLAC Laboratory Animal Co., Ltd. (Shanghai, China). All mice were housed in specific pathogen free (SPF) grade environment with 12 h light/dark cycle and free access to food and water.

### Platelet preparation

All experimental procedures involving collection of human and mouse blood were approved by the Ethic Committee of Xuzhou Medical University. Informed consent has been obtained from all participants. Platelets were prepared from human and mouse blood as described previously [18, 19]. For human platelets, venous blood was collected into a tube anti-coagulated with trisodium citrate, glucose and citric acid (ACD) and centrifuged for 20 min at 120×*g* at room temperature to obtain platelet-rich plasma (PRP). Platelet pellets were then obtained by centrifugation of PRP at 1350×*g* for 15 min, followed by washing three times in CGS buffer and resuspended in Tyrode’s buffer. Mouse platelets were isolated from ACD anti-coagulated blood, washed using CGS buffer and resuspended in Tyrode’s buffer.

### Treatment of platelets with PD

Isolated human platelets were incubated with different concentrations of PD (0, 1, 10 and 20 μM) at 37 °C for 30 min followed by measuring relevant parameters.

### Platelet aggregation

Platelet aggregation was performed using human washed platelets in the presence of fibrinogen (0.5 mg/ml). After PD treatment, platelet aggregation in response to collagen (2.5 μg/ml), ADP (5 μΜ), arachidonic acid (250 μg/ml) or epinephrine (75 μΜ) was evaluated in a light transmittance aggregometry (Helena Aggram, Helena Laboratories, Beaumont, USA) as previously described [18–20]. Platelet aggregation was quantified as the percentage of maximum platelet aggregation (monitored for 5 min) in the absence of drug.

### Platelet activation

Platelet activation was assessed through measuring the surface expression of the α-granule glycoprotein, P-selectin, and by the activation-dependent binding of PAC-1 to platelet α_IIb_β_3_ as described previously [20]. Briefly, after incubated with different concentrations of PD, human platelets were stimulated with collagen (10 μg/ml) or ADP 10 (μM) in the presence of PE-conjugated anti-P-selectin antibody and FITC-conjugated PAC-1 antibody followed by analyzing platelet activation by flow cytometry. P-selectin expression and PAC-1 binding were defined as the percentage of platelets in platelet-specific gate with positive staining of anti-P-selectin antibody and PAC-1 antibody.

### Surface expression of platelet receptors

After treatment of human platelets with different doses of PD, FITC-conjugated anti-CD42b antibody (GPIbα), FITC-conjugated mouse anti-human CD41a antibody (α_IIb_) or anti-human GPVI antibody (detected by FITC-conjugated goat anti-mouse IgG) were added and incubated for 30 min at room temperature followed by flow cytometry analysis of the expression of platelet receptors. For measuring mouse platelet receptor expression, FITC-conjugated anti-mouse CD41a antibody was used.

### Platelet spreading

Human platelets were placed on fibrinogen-coated glass coverslips (20 μg/ml fibrinogen, 4 °C overnight) at 37 °C for 90 min followed by washing with PBS. After that, platelets were fixed, permeabilized, stained with Alexa Fluor-546-labelled phalloidin and observed by a fluorescence microscopy (Nikon-80i) using an X100 oil objective. The surface coverage was quantified using Image J software.

### Clot retraction

Washed human platelets were supplemented with 2 mM Ca^2+^ and 0.5 mg/ml fibrinogen and clot retraction was induced by thrombin (1 U/ml) treatment at 37 °C as described previously [19]. Images were captured every 30 min.

### Tail bleeding assay

Mouse platelets (1 × 10^8^) were treated with PD (20 μM) or vehicle at 37 °C for 30 min and infused into mice made thrombocytopenic by intraperitoneal injection of rat anti-mouse αIIb antibody (MWReg 30) at 0.1 mg/kg body weight as described previously [19] followed by measuring tail bleeding time [18].

### FeCl_3_-induced arterial thrombosis

After treated with PD (20 μM) or vehicle at 37 °C for 30 min, mouse platelets were labelled with calcein and infused into wild-type mice via tail vein injection. 10% FeCl_3_ was used to cause damages to mesenteric arterioles and thrombus formation was monitored by a fluorescence microscopy (Olympus BX53) [19].

### Western blotting

Washed human platelets were treated with different concentrations of PD followed by measuring the expression of α_IIb_β_3_, GPIbα and GPVI by western blot using rabbit polyclonal CD41/Integrin Alpha 2b Antibody (Proteintech, Rosemont, IL, USA), mouse monoclonal CD42b antibody (SZ2) (Santa Cruz, Dallas, Texas, USA) or GP6 Monoclonal Antibody (HY101) (Ebioscience, San Diego, CA, USA) respectively. For some experiments, washed platelets were treated with CRP (5 μg/ml) or thrombin (1 U/ml) in the presence different concentrations of PD or vehicle for 15 min. Levels of total and phosphorylated Syk (anti-Tyr-525 and pan-Syk, Bioworld Technology) and PLCγ2 (anti-Tyr-1217 and pan-PLCγ2; Bioworld Technology) were assessed by SDS-PAGE/western blot.

### Platelet apoptosis

Platelet apoptosis was measured as previously described [21]. In brief, washed human platelets were treated with different doses of PD followed by measuring the surface expression of Annexin-V by flow cytometry using Annexin V Apoptosis Detection Kit (eBioscience, San Diego, CA, USA) or cleaved caspase-3 expression by western blot. Cleaved caspase-3 expression (rabbit monoclonal antibody against caspase-3, Cell Signaling Technology) was analyzed using Image J software and presented as a ratio relative to full length caspase-3.

### Statistical analysis

Data are represented as mean ± standard deviation (SD). One-way ANOVA was conducted for comparison of difference among different groups. Two-way ANOVA with Bonferroni post-tests was performed for comparison among different groups over time. P < 0.05 indicates statistically significance.

## Results

### Impaired platelet aggregation after PD treatment

**Fig. 1:**
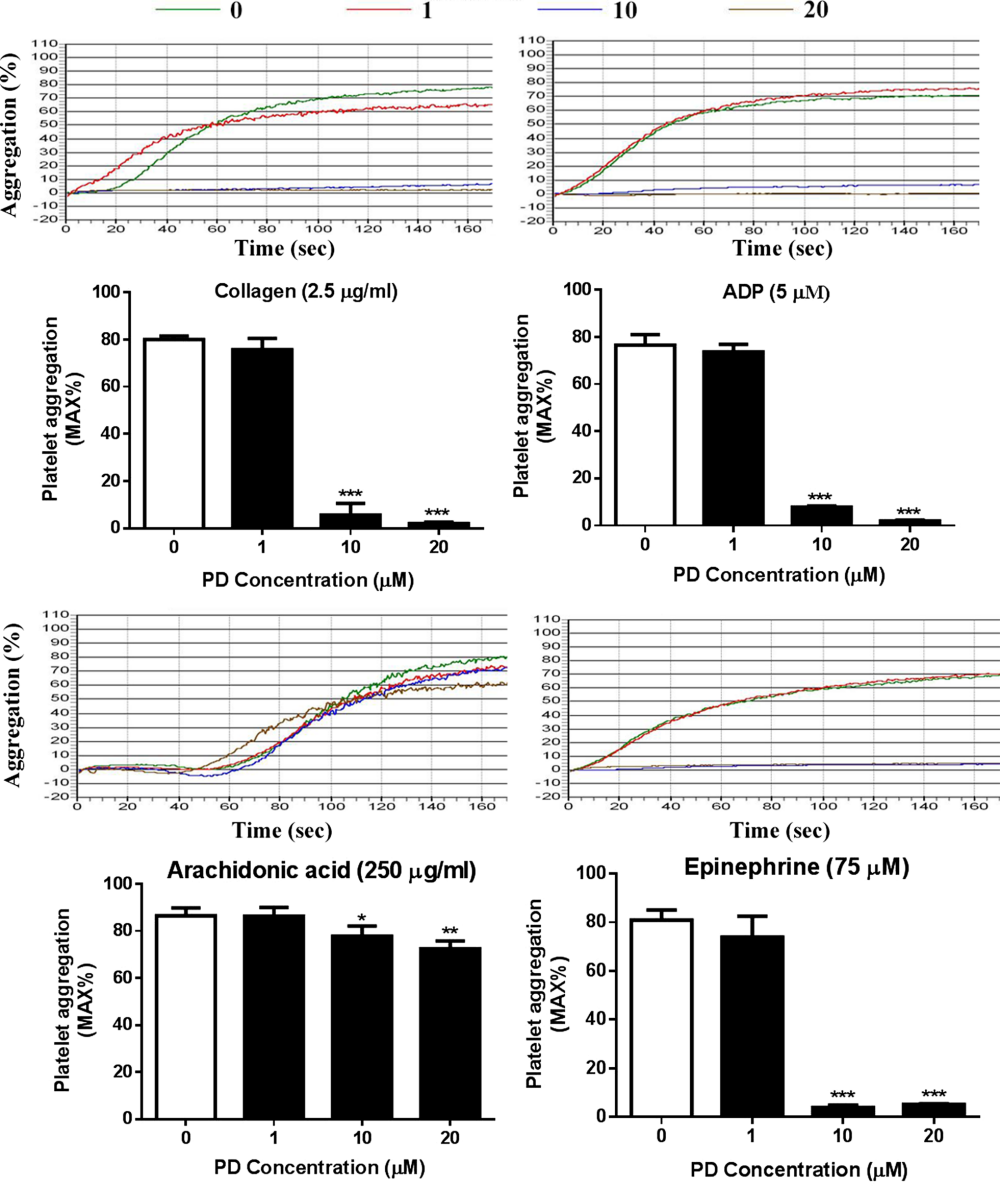
Platelet aggregation after PD treatment. Human washed platelets were treated with different concentrations of PD (0, 1, 10, 20 μM) at 37 °C for 30 min followed by measuring platelet aggregation in response to collagen (2.5 μg/ml), ADP (5 μM), arachidonic acid (250 μg/ml) and epinephrine (75 μM) in a light transmittance aggregometry (mean ± SD, n = 4). Compared with 0, *P < 0.05; **P < 0.01; ***P < 0.001

Platelet aggregation plays an important role in thrombosis and hemostasis and is a reliable marker to evaluate platelet function [22]. To evaluate whether PD affects platelet function, human platelets were isolated and platelet aggregation in response to agonist stimulation was performed in a light transmittance aggregometry. As seen in Fig. 1, PD-treated (10 and 20 μM) platelets displayed a significant reduction of platelet aggregation in response to collagen (2.5 μg/ml), ADP (5 μM), arachidonic acid (250 μg/ml) or epinephrine (75 μM) compared to vehicle-treated platelets. However, the low dose of PD (1 μM) did not affect platelet aggregation.

### Reduced activation of PD-treated platelets

**Fig. 2:**
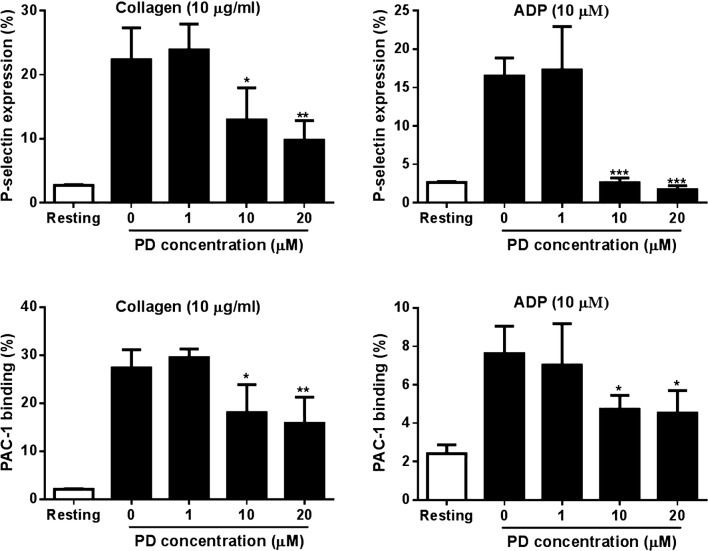
Platelet P-selectin expression and aIIbb3 activation. After PD treatment, human platelets were stimulated with collagen (10 μg/ml) or ADP 10 (μM) followed by measuring platelet surface P-selectin expression by flow cytometry using PE-conjugated anti-P-selectin antibody and FITC-conjugated PAC-1 antibody (mean ± SD, n = 3). Compared with 0, *P < 0.05; **P < 0.01; ***P < 0.001

As platelet activation is the prerequisite for platelet aggregation and subsequent thrombus formation, we next investigated the effect of PD on platelet activation in response to agonist stimulation through measuring the surface expression of P-selectin, a platelet activation marker and PAC-1 binding, an integrin α_IIb_β_3_ activation marker by flow cytometry. Consistent with impaired platelet aggregation, platelets treated with PD (10 and 20 μM) showed significantly reduced surface expression of P-selectin as well as PAC-1 binding in response to collagen (10 μg/ml) or ADP (10 μM) stimulation (Fig. 2), suggesting PD inhibits agonist-induced platelet activation.

### PD inhibits integrin aIIbb3 outside-in signaling

**Fig. 3:**
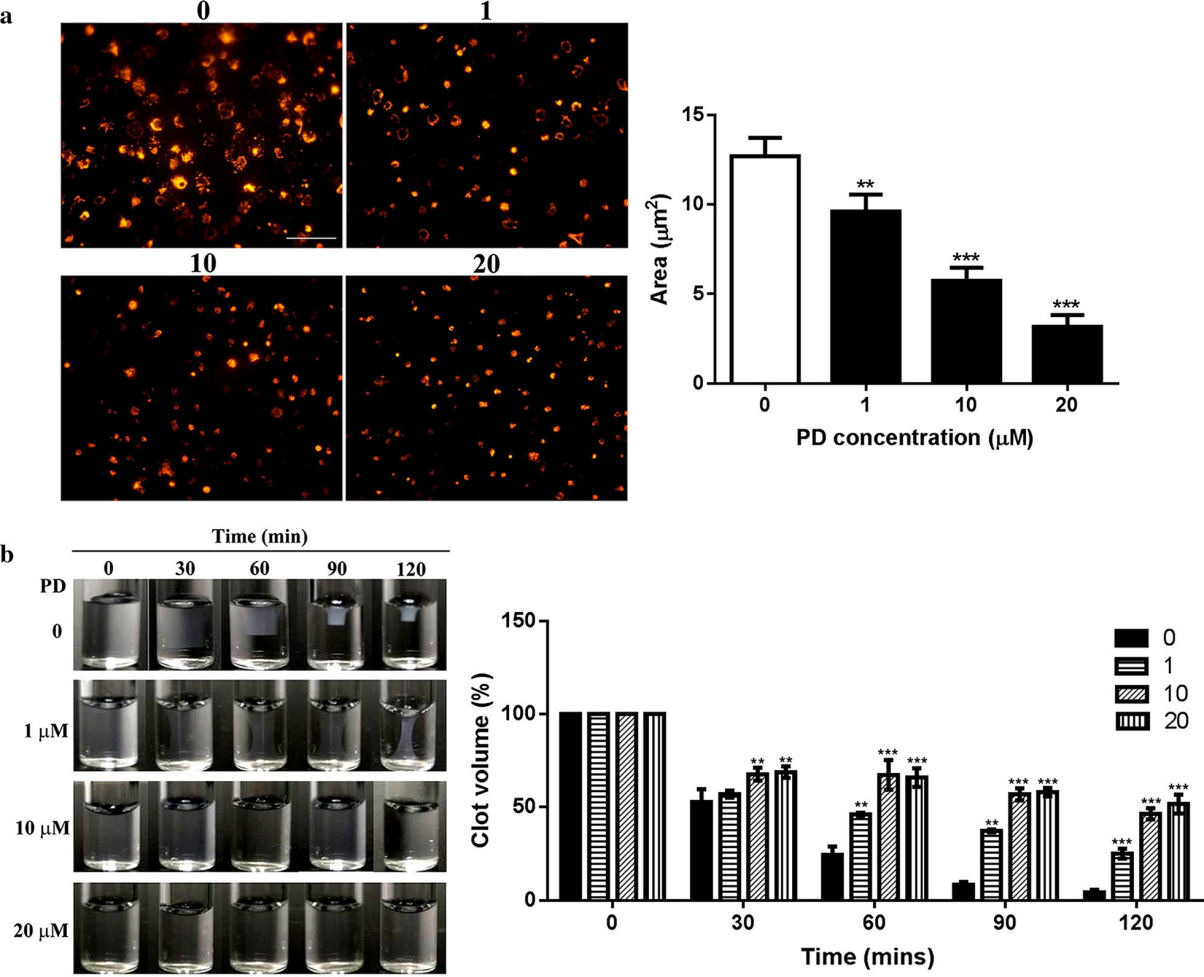
Platelet spreading and clot retraction. **a** After PD treatment, washed human platelets were placed on fibrinogen-coated glass coverslips and allowed to spread at 37 °C for 90 min followed by staining with Alexa Fluor-546-labelled phalloidin and subsequent observation under a fluorescent microscopy (mean ± SD, n = 3). **b** PD-treated platelets were supplemented with 2 mM Ca^2+^ and 0.5 mg/ml fibrinogen and clot retraction was induced by thrombin (1 U/ml) treatment at 37 °C. Images were captured every 30 min (mean ± SD, n = 3). Compared with 0, **P < 0.01; ***P < 0.001

To investigate the effect of PD on platelet α_IIb_β_3_ outside-in signaling transduction, we measured platelet spreading on immobilized fibrinogen, an event regulated by early-α_IIb_β_3_ outside-in signaling [5, 23]. As seen in Fig. 3a, PD inhibits platelet spreading in a dose dependent manner as the surface coverage area of spread platelets on fibrinogen after PD treatment was significantly smaller than platelets after vehicle treatment, with a smallest coverage area being observed in platelets treated with 20 μM PD. Meanwhile, we also assessed platelet-mediated clot retraction, another process regulated by late-α_IIb_β_3_ outside-in signaling [5, 23]. Consistent with the profile of platelet spreading, clot retraction in PD-treated platelets was significantly inhibited in a dose-dependent manner as the clot volume of PD-treated platelets was significantly higher than vehicle-treated platelets (Fig. 3b). Taken together, these data demonstrate that PD inhibits platelet aIIbb3 outside-in signaling transduction.

### Decreased phosphorylation of Syk and PLCγ2 in PD-treated platelets

**Fig. 4:**
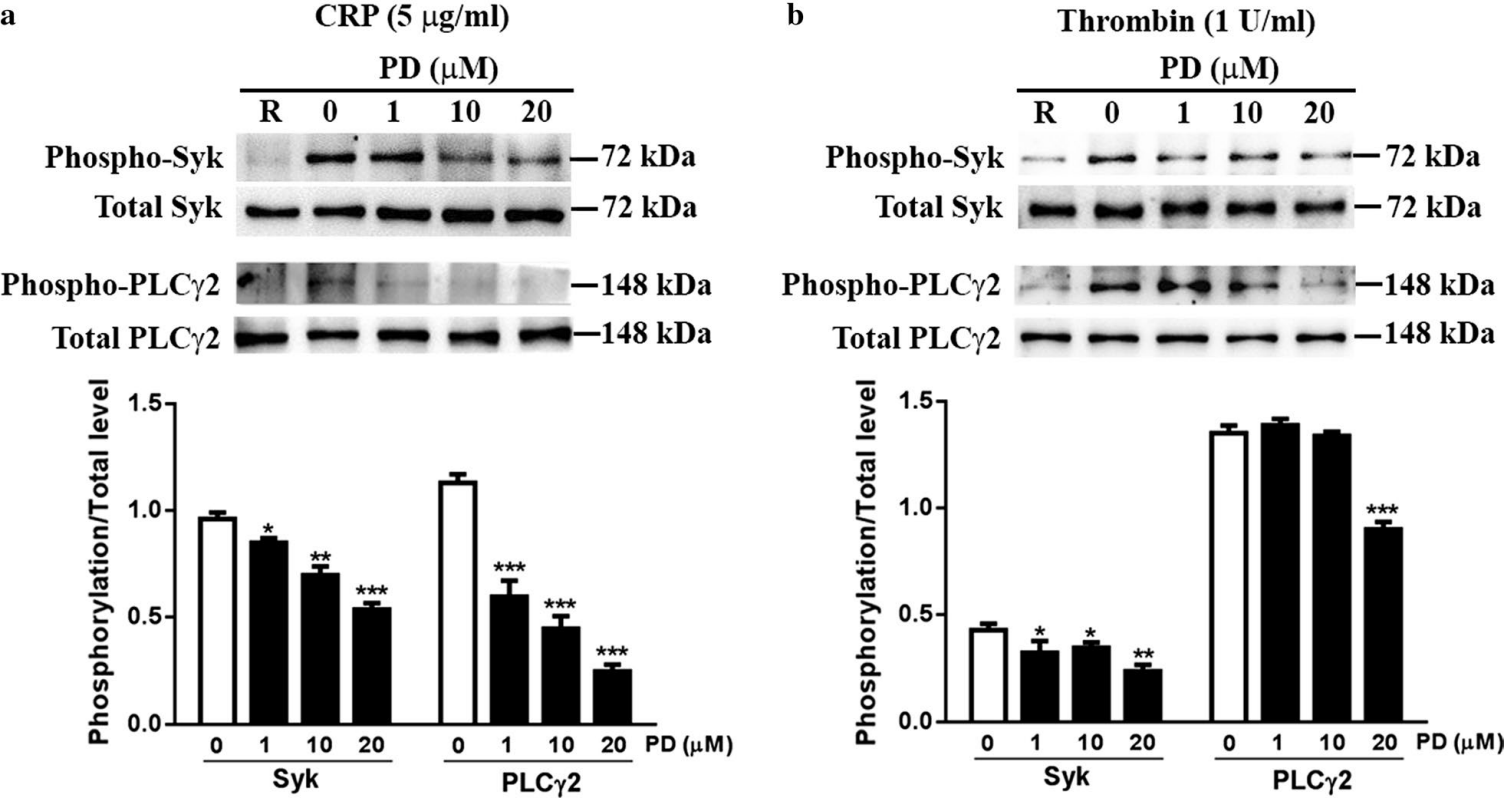
Phosphorylation level of Syk and PLCγ2. PD-treated human washed platelets were stimulated with 5 μg/ml CRP (**a**) or 1 U/ml thrombin (**b**) for 15 min and the phosphorylation level of Syk and PLCγ2 was measured by western blot. The protein expression was quantified using Image J software and represented as a ratio of phosphorylation to the total level (mean ± SD, n = 3). Compared with 0, *P < 0.05; **P < 0.01; ***P < 0.001

As PD has been demonstrated to affect platelet activation and aggregation, we next evaluated the effect of PD on platelet intracellular signaling transduction through measuring the phosphorylation level of Syk and PLCγ2, which have been demonstrated to play important roles in platelet function and aIIbb3 signaling transduction [5]. As seen in Fig. 4a, PD-treated platelets displayed significantly impaired phosphorylation of Syk and PLCγ2 after collagen-related peptide (CRP) in a dose dependent manner. As thrombin-mediated clot retraction was impaired after PD treatment, we next measured the phosphorylation level of Syk and PLCγ2 which have been showed to be involved in the regulation of clot retraction [24] and showed reduced phosphorylation of Syk and PLCγ2 in platelets after thrombin stimulation (Fig. 4b). These data indicated that PD decreases aIIbb3 outside-in signaling transduction through downregulation of the phosphorylation of Syk and PLCγ2.

### PD-treated platelets display impaired hemostasis and arterial thrombosis in vivo

**Fig. 5:**
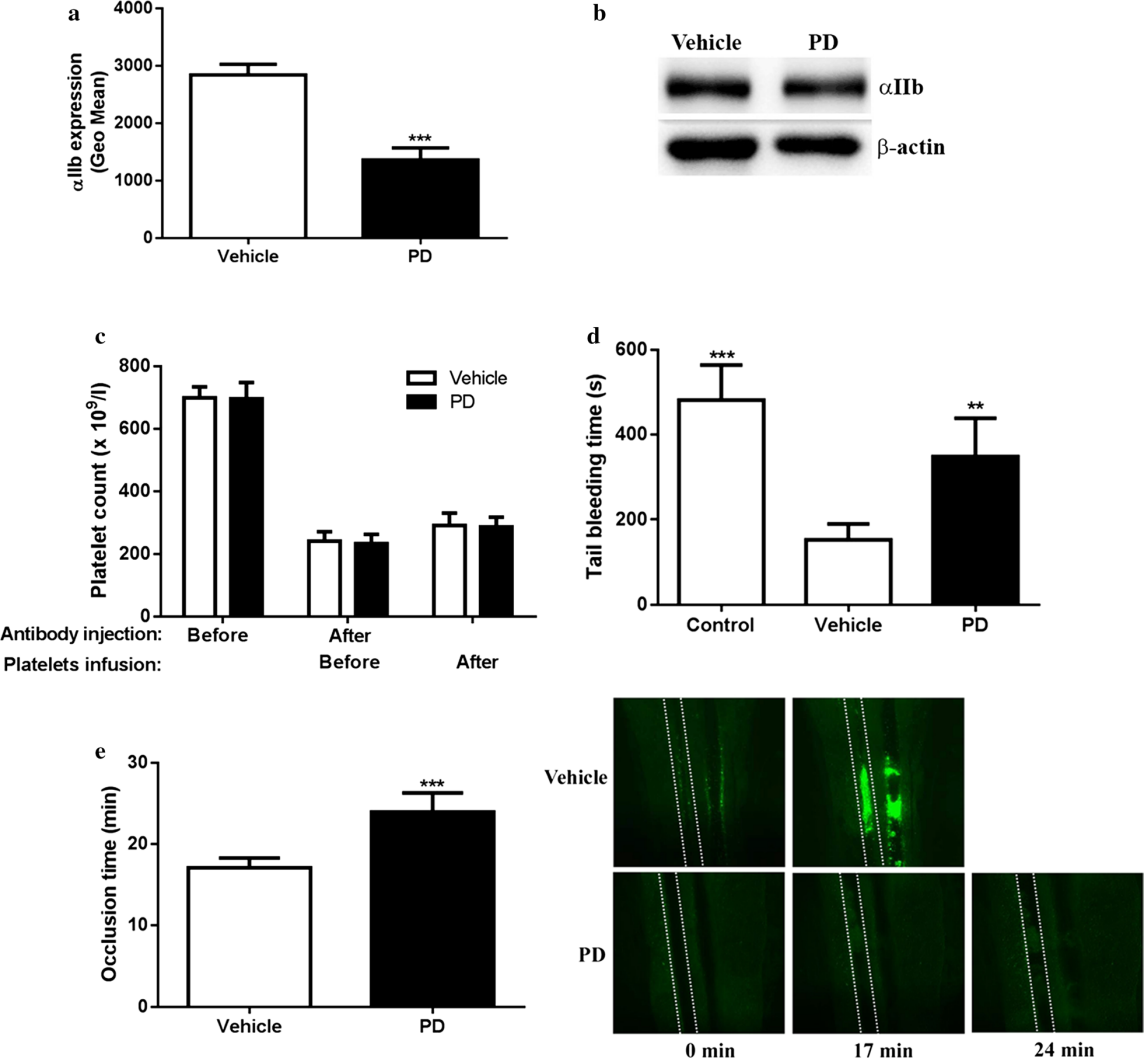
The effect of PD on mouse platelet aIIbb3 expression and function. Washed mouse platelets were treated with PD (20 μM) followed by measuring aIIb expression by flow cytometry (mean ± SD, n = 3) (**a**) and western blot (**b**). PD or vehicle-treated mouse platelets were infused into thrombocytopenic mice or wild-type mouse followed by measuring platelet count (**c**) and tail bleeding time (mean ± SD, n = 6) (**d**) or arterial thrombus formation (mean ± SD, n = 6) (**e**) respectively. Comparison between Vehicle and PD, **P < 0.01; Comparison between Control and Vehicle, ***P < 0.001. Control in **d** indicates platelet depleted mice without transfusing WT platelets

Platycodin D (5, 20 and 100 mg/kg) was intraperitoneally injected into mice to evaluate the effect of PD on platelet function in vivo and we found PD-injected mice displayed weakness of limbs, depression as well as a large amount of ascites without abnormalities of other organs within 30 min after administration. However, low dose of PD (1 mg/kg) treatment did not induce such side effects mentioned above, but significantly prolonged tail bleeding time (182.8 ± 58.9 s vs 70.4 ± 20.9 s for vehicle, mean ± SD, n = 5) (P = 0.004) and delayed in vivo arterial thrombus formation (67.8 ± 13.7 min vs 14.5 ± 3.9 min for vehicle, mean ± SD, n = 5) (P = 0.0003) compared with vehicle treatment. To exclude other factors which might affect platelet function in vivo after PD injection, mouse platelets were treated with PD ex vivo and infused into wild-type mice followed by measuring tail bleeding and arterial thrombus formation to investigate the specific effect of PD on platelet function in vivo. Firstly, to confirm whether PD exerts the same effects on the surface expression of mouse platelet receptor, mouse platelets were treated with PD followed by measuring the surface expression of α_IIb_β_3_ by flow cytometry. As seen in Fig. 5a, a significantly reduced surface expression of α_IIb_β_3_ was observed in PD-treated platelets compared with vehicle. However, western blot analysis of platelet lysates showed PD treatment did not induce the change of α_IIb_β_3_ expression (Fig. 5b), indicating PD also induces the internalization of mouse platelet receptor aIIbb3. To exclude the effect of endogenous platelets on hemostasis, we performed adoptive transfer of PD-treated platelets into thrombocytopenic wild-type mice which were made through intraperitoneal injection of rat anti-mouse α_IIb_ antibody (MWReg 30). Prior to analysis of tail bleeding and arterial thrombus formation, we measured the peripheral platelet count before or after administration of anti-mouse α_IIb_ antibody (9 h post injection) as well as after adoptive transfer (0.5 h post transfusion) and showed platelet count was dramatically reduced after injection of MWReg 30 and recovered after adoptive transfer (Fig. 5c), indicating transfused platelets were indeed circulating in the peripheral blood rather than being removed by residual anti-mouse α_IIb_ antibody. In addition, no differences of the platelet count in mice receiving either vehicle- or PD-treated platelets were observed. However, thrombocytopenic wild-type mice receiving infusion of PD-treated platelets displayed a significantly prolonged tail bleeding time compared with those receiving infusion of vehicle-treated platelets (P < 0.01) (Fig. 5d). In addition, we also assessed whether PD affects arterial thrombosis in vivo using FeCl_3_-induced arterial thrombus formation model and showed PD treatment impaired arterial thrombosis as demonstrated by significantly prolonged occlusion time (P < 0.001) (Fig. 5e). These findings indicate that PD induces internalization of mouse platelet receptor α_IIb_β_3_ and impairs hemostasis and *thrombosis* in vivo.

### PD does not induce platelet apoptosis

**Fig. 6:**
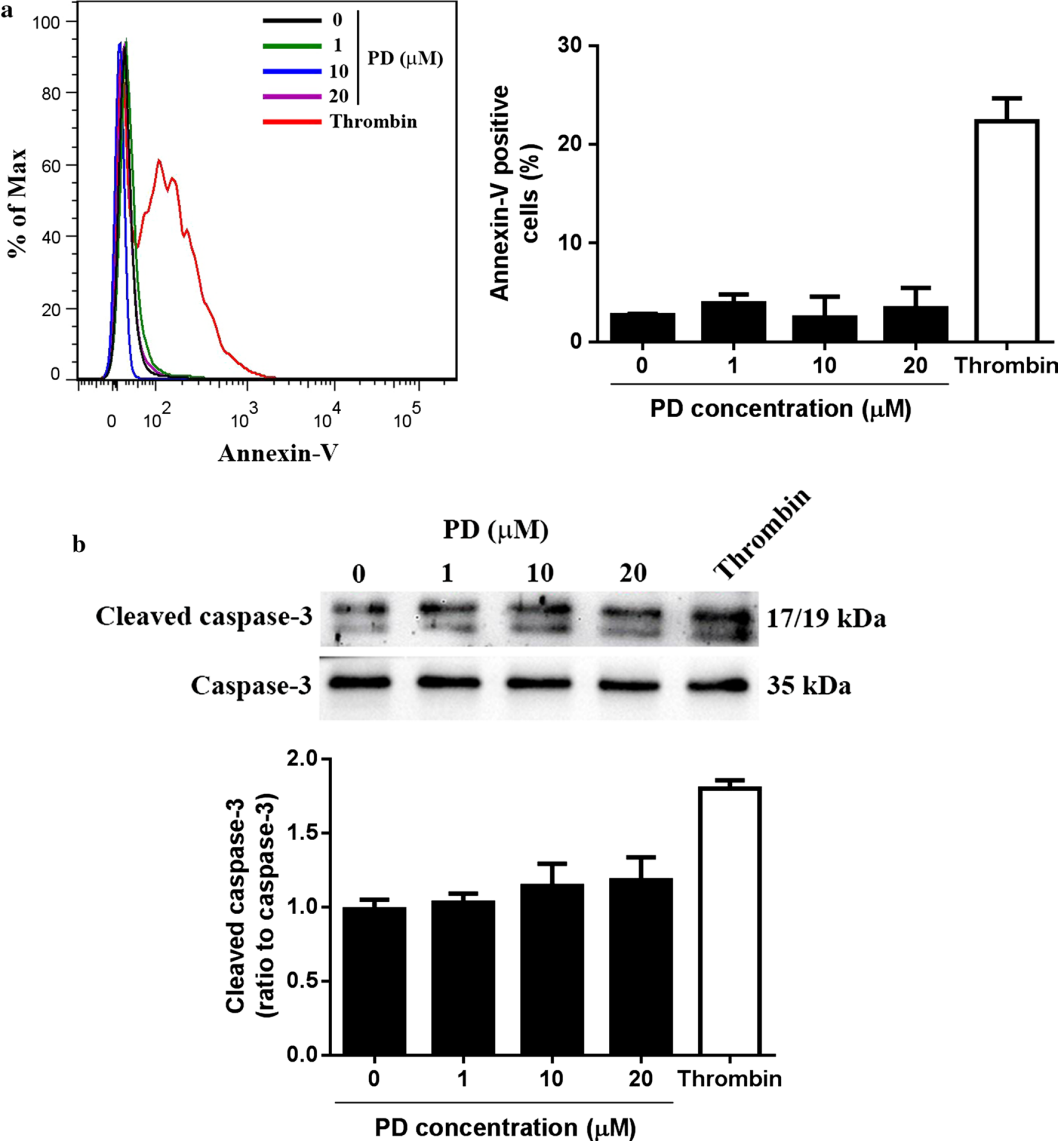
Analysis of platelet apoptosis after PD treatment. Washed platelets were treated with different doses of PD followed by measuring the surface expression of Annexin-V by flow cytometry (mean ± SD, n = 4) (**a**) and cleaved caspase-3 by western blot (mean ± SD, n = 4) (**b**). Thrombin (1 U/ml) stimulation was used as a positive control. Representative flow cytometry and western blot image was shown from four independent experiments

Previous studies demonstrated that PD exerts anti-tumor effects through inducing cell apoptosis [13, 25, 26]. To evaluate whether PD induces platelet apoptosis, isolated human platelets were incubated with different concentrations of PD followed by measuring Annexin-V expression by flow cytometry. Different to its effect on tumor cells, PD did not trigger platelet apoptosis as no significant increase of Annexin-V expression was observed in platelets even after treated with a higher dose of PD (20 μM) (Fig. 6a), suggesting PD does not induce platelet apoptosis. Consistently, no significant increase of cleaved caspase-3 expression was observed in PD-treated platelets (Fig. 6b).

### PD induces internalization of glycoprotein receptors

**Fig. 7:**
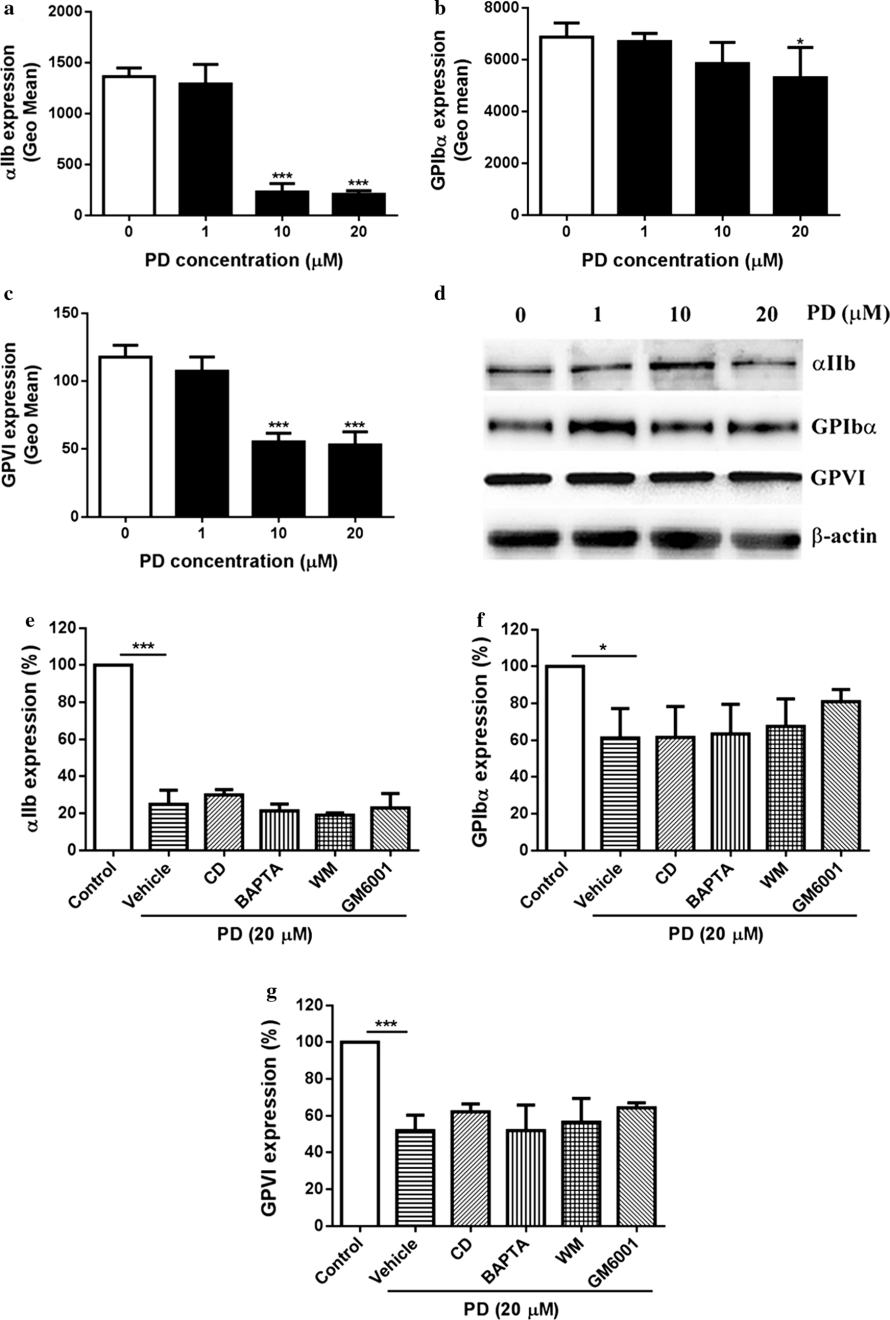
Expression of platelet glycoprotein receptors α_IIb_β_3_, GPIbα and GPVI. After PD treatment, the expression of platelet α_IIb_β_3_, GPIbα and GPVI was measured by flow cytometry (mean ± SD, n = 3) (**a**–**c**) and western blot (**d**). Prior to PD treatment, washed platelets were treated with cytochalasin D (CD) (20 μM), BAPTA-AM (BAPTA) (20 μM), wortmannin (WM) (10 μM) or GM6001 (100 μM) followed by measuring the surface expression of platelet α_IIb_β_3_, GPIbα and GPVI by flow cytometry (mean ± SD, n = 3) (**e**–**g**). Compared with 0, *P < 0.05; ***P < 0.001

Platelet glycoprotein receptors GPIbα, GPVI and α_IIb_β_3_ have been demonstrated to play critical roles in the regulation of platelet activation, aggregation and thrombus formation [27, 28]. To uncover the molecular mechanism by how PD affects platelet function, we measured the surface expression of platelet integrin α_IIb_β_3_, GPIbα and GPVI by flow cytometry and western blot. Interestingly, we found significantly reduced surface expression levels of α_IIb_β_3_, GPIbα and GPVI in PD-treated platelets compared with those in vehicle-treated platelets (Fig. 7a–c), suggesting PD downregulates the surface expression of these platelet glycoprotein receptors. To assess whether PD’s effect is temporary, we monitored the surface expression of glycoprotein receptors after PD treatment over time and found no recovery of the reduced surface expression of receptors (data not shown), indicating PD’s effect on the expression of platelet glycoprotein receptors is permanent. To further investigate whether the reduced surface expression was due to internalization as PD has been reported to interfere with cytoskeleton assembly [29, 30], we performed western blot analysis of platelet lysates after PD treatment using antibodies against the extracellular domain of α_IIb_β_3_, GPIbα and GPVI and found PD treatment did not alter changes of the expression of these receptors even at a higher dose (Fig. 7d) without detectable lower molecular weight band (data not shown), suggesting PD triggers internalization of platelet glycoprotein receptors. To evaluate whether PD triggers metalloproteinase-mediated shedding of platelet surface receptors, we added broad range metalloproteinase inhibitor GM6001 to platelets under PD treatment and found it did not prevent PD-induced downregulation of the surface expression of platelet glycoprotein receptors even a slight increase of GPIbα surface expression was observed (Fig. 7e–g), which might be explained by metalloproteinase-mediated constitutive shedding of GPIbα.

As previous studies showed that platycodin D alters cytoskeleton through targeting actin [30] or microtubule [29], we used cytochalasin D (an inhibitor of actin polymerization) [31], BAPTA-AM (membrane permeable calcium chelator with microtubule depolymerizing activity) [32] and wortmannin (an inhibitor of myosin light chain kinase) [33] to assess whether they are able to inhibit the internalization of platelet receptors after PD treatment. Unfortunately, all these reagents could not inhibit the internalization of platelet α_IIb_β_3_, GPIbα and GPVI (Fig. 7e–g). Surprisingly, a slightly increase of α_IIb_β_3_ surface expression in PD-treated platelets after addition of cytochalasin D.

## Discussion

As a triterpenoid saponin, platycodin D is one of the major bioactive components of the roots of *P. grandiflorum,* a traditional Chinese medicinal herb [7, 8]. Several studies have shown that platycodin D affects cell biological behaviors through exerting multiple functions, such as anti-nociceptive [10], antiviral [11], anti-inflammatory [12], anti-cancer [13], immunomodulatory [14, 15], and hepatoprotective activities [8]. However, whether platycodin D plays a role in platelet function and thrombus formation remains poorly understood. In this study, we demonstrated that Platycodin D inhibits platelet activation, aggregation, α_IIb_β_3_ signaling transduction and impairs in vivo hemostasis and thrombosis.

In recent years, platycodin D in the field of cancer treatment has received a special attention as a potential anti-cancer compound. Several in vitro and in vivo studies demonstrated that platycodin D displays a broad spectrum of cytotoxicity against a wide range of human cancer cell lines through multiple mechanisms, such as inhibition of cell proliferation and survival, induction of apoptosis, autophagy, cell cycle arrest, inhibition of angiogenesis and metastasis as well as regulation of transcription factors [13]. In addition to its effect alone on tumor cells, platycodin D has also been showed to display enhanced anti-tumor effects in combination with other anti-tumor drugs, such as doxorubicin, osthol [34, 35]. Different to its effect on inducing apoptosis of tumor cells, in this study, we showed that platycodin D does not trigger platelet apoptosis as demonstrated by no increased expression of annexin-V in platycodin D-treated platelets. However, platycodin D treatment significantly inhibits platelet aggregation, activation, spreading on immobilized fibrinogen and clot retraction. In addition, the phosphorylation level of Syk and PLCγ2, which are involved in the regulation of platelet intracellular signaling transduction [5], was also significantly reduced in platycodin D-treated platelets after CRP or thrombin stimulation. Furthermore, platycodin D-treated platelets displayed significantly impaired in vivo hemostasis and arterial thrombus formation. Taken together, these data show that platycodin D inhibits platelet function and thrombosis independent of platelet apoptosis, suggesting platycodin D might also be a potential anti-thrombotic drug rather than just an anti-cancer compound.

The main function of platelets is to participate in thrombosis and hemostasis and platelet surface glycoprotein receptors, such as α_IIb_β_3_, GPIbα and GPVI, play a critical role in the regulation of platelet function [28]. In case of vascular vessel injury, platelet glycoprotein receptor GPIbα and GPVI adhere to the damaged vessel through recognition of exposed VWF and collagen respectively followed by transduction of intra-platelet signaling pathway and subsequent activation of α_IIb_β_3_, which binds to fibrinogen or VWF and mediates platelet aggregation and thrombus formation [2, 28]. Given inhibition of platelet aggregation and thrombosis by platycodin D, plus the importance of platelet glycoprotein receptors α_IIb_β_3_, GPIbα and GPVI in platelet function, we measured the surface expression of these receptors after platycodin D treatment and found significantly decreased surface expression of these receptors in platycodin D-treated platelets, suggesting platycodin D inhibits platelet function through downregulation of platelet surface glycoprotein receptors.

A cytoskeleton is present in all cells as a system of intracellular filaments and plays an important role in the regulation of cell physiology from several aspects, including cell shape, mitosis, cell division, cell polarity and extracellular matrix patterning [36, 37]. Consisted of actin, microtubules and septins, the cytoskeletal networks have been demonstrated to be involved in several cellular signaling pathways, such as uptake of extracellular material (endocytosis) [38], intracellular transport [39], and chromosomes segregation during cellular division [40]. A previous study showed that platycodin D induced microtubule polymerization, mitotic arrest and polyploidy in leukemia cells, leading to endoreduplication, inhibition of cell proliferation and promotion of cell apoptosis [29]. In addition, treatment of B16F10 cell (a melanoma cell line) with platycodin D in vitro resulted in less organized actin fibres as well as reduced filopodia and lamellipodia formation in a dose dependent manner [30]. Considering the effect of platycodin D on cytoskeleton, we hypothesized that platycodin D-mediated downregulation of the surface expression of platelet glycoprotein receptors might be attributed to internalization. To test that, we performed western blot analysis of platelet lysates using antibodies against the extracellular domain of platelet glycoprotein receptors and showed no changes of the expression of platelet receptors α_IIb_β_3_, GPIbα and GPVI after platycodin D treatment even at a higher dose, confirming that platycodin D induces internalization of platelet glycoprotein receptors. As platycodin D alters cytoskeleton through targeting actin or microtubule [29, 30], we used cytochalasin D (an inhibitor of actin polymerization), BAPTA-AM (with microtubule depolymerizing activity) and wortmannin (an inhibitor of myosin light chain kinase) to evaluate whether these reagents inhibits platycodin D-mediated internalization of receptors. Surprisingly, none of these agents can block the internalization, suggesting other factors might be involved in the internalization induced by platycodin D.

## Conclusion

Our study demonstrates that platycodin D inhibits platelet activation, aggregation, in vivo hemostasis and arterial thrombus formation through inducing the internalization of platelet glycoprotein receptors α_IIb_β_3_, GPIbα and GPVI, suggesting it might be used as a potential anti-thrombotic drug.

## Abbreviations

ADP: adenosine diphosphate
PRP: platelet-rich plasma
PD: platycodin D
PLCγ2: phospholipase cγ2
syk: spleen tyrosine kinase
